# DegS and RseP Homologous Proteases Are Involved in Singlet Oxygen Dependent Activation of RpoE in *Rhodobacter sphaeroides*

**DOI:** 10.1371/journal.pone.0079520

**Published:** 2013-11-05

**Authors:** Aaron M. Nuss, Fazal Adnan, Lennart Weber, Bork A. Berghoff, Jens Glaeser, Gabriele Klug

**Affiliations:** 1 Institute of Microbiology and Molecular Biology, Giessen University, Giessen, Germany; 2 Department of Molecular Infection Biology, Helmholtz Centre for Infection Research, Braunschweig, Germany; 3 Department of Cell and Molecular Biology, Biomedical Center, Uppsala University, Uppsala, Sweden; Arizona State University, United States of America

## Abstract

Singlet oxygen (^1^O_2_) is the main agent of photooxidative stress and is generated by photosensitizers as (bacterio)chlorophylls. It leads to the damage of cellular macromolecules and therefore photosynthetic organisms have to mount an adaptive response to ^1^O_2_ formation. A major player of the photooxidative stress response in *Rhodobacter sphaeroides* is the alternative sigma factor RpoE, which is inactivated under non-stress conditions by its cognate anti-sigma factor ChrR. By using random mutagenesis we identified RSP_1090 to be required for full activation of the RpoE response under ^1^O_2_ stress, but not under organic peroxide stress. In this study we show that both RSP_1090 and RSP_1091 are required for full resistance towards ^1^O_2_. Moreover, we revealed that the DegS and RseP homologs RSP_3242 and RSP_2710 contribute to ^1^O_2_ resistance and promote ChrR proteolysis. The RpoE signaling pathway in *R. sphaeroides* is therefore highly similar to that of *Escherichia coli*, although very different anti-sigma factors control RpoE activity. Based on the acquired results, the current model for RpoE activation in response to ^1^O_2_ exposure in *R. sphaeroides* was extended.

## Introduction

Light and oxygen in combination with a photosensitizer lead to the formation of toxic singlet oxygen (^1^O_2_). The photosensitizer absorbs light and transfers energy to molecular oxygen, causing a spin conversion of an electron, thereby forming the highly reactive ^1^O_2_ [1]. Excess of ^1^O_2_ is toxic for the cell, as it can react with macromolecules like proteins, lipids and nucleic acids [2,3]. The cell needs to respond to this so called photooxidative stress to prevent cellular damages which consequently would lead to cell death. 

Facultative photosynthetic α-proteobacteria like *Rhodobacter sphaeroides* induce the formation of the photosynthetic apparatus when the oxygen tension in the environment decreases. The synthesized bacteriochlorophyll molecules and their precursors can act as potent cellular photosensitizers. Nevertheless, even when photosynthetic pigments are highly abundant in the cell, *R. sphaeroides* grows well in the presence of light and oxygen. The presence of carotenoids protects against ^1^O_2_ caused damages and in addition, *R. sphaeroides* mounts a molecular response to ^1^O_2_ exposure, which is independent of carotenoids [4,5]. This response partly depends on the alternative group IV sigma factor RpoE. RpoE is inactivated by forming a stable complex with its cognate anti-sigma factor ChrR in a 1:1 stoichiometry [6,7]. When *R. sphaeroides* cells are exposed to ^1^O_2_, the RpoE:ChrR complex dissociates, RpoE binds to the RNA polymerase and induces the expression of target genes [4,6]. When the crystal structure of the RpoE:ChrR complex was solved it was shown that the zinc containing anti-sigma domain (ASD) of ChrR is necessary for the interaction with RpoE [7]. The ASD is conserved in many bacterial anti-sigma factors [7]. A second zinc containing ChrR domain, the cupin like domain (CLD), is necessary for activation of RpoE by ^1^O_2_. It was proposed that amino acid side chains or a ligand in the ChrR-CLD are targets of unknown chemical modification by ^1^O_2_ that lead to dissociation of the RpoE:ChrR complex [7]. The CLD could also play a role in promoting an association of the RpoE:ChrR complex with the photosynthetic membrane, the main source of ^1^O_2_ generation [8]. 

In bacteria one mechanism of sigma factor activation is the proteolysis of the cognate anti-sigma factor. In the Gram negative bacterium *Escherichia coli*, the alternative sigma factor σ^E^ (also known as RpoE) is inactivated by the binding of its cognate anti-sigma factor RseA, which is membrane localized. Under cell envelope stress conditions, RseA is stepwise proteolyzed, thus RpoE is released and can bind to the RNA polymerase [9]. Interestingly, the N-terminal ASD of ChrR and RseA are similar in structure, but not in amino acid sequence [7,10].

Homologs of the RpoE:ChrR complex can be found in many α-, β- and γ-proteobacteria [11]. In the α-proteobacterium *Caulobacter crescentus* RpoE activity is not only induced by ^1^O_2_, but also by exposure to organic peroxide (tert-butyl-hydroperoxide, tBOOH), cadmium and UV-A irradiation [12]. Specific amino acid residues in the anti-sigma factor ChrR may be required for the specific response to either ^1^O_2_, organic peroxide and UV-A irradiation or cadmium [12]. 

The *R. sphaeroides* RpoE regulon is well defined, but the exact mechanism of RpoE:ChrR dissociation is still unknown. Recent work reported that the anti-sigma factor ChrR is degraded in the presence of ^1^O_2_ and tBOOH [13,14], but the proteases involved in ChrR proteolysis are yet unknown. This motivated us to search for factors that are involved in RpoE activation under photooxidative stress. A Tn5 mutagenesis of the *R. sphaeroides* wild type revealed that insertion of Tn5 into the RSP_1090 generated a strain highly sensitive to ^1^O_2_. Consequently, we investigated the impact of genes encoded in the RSP_1091-1087 operon in the photooxidative stress response and showed that RSP_1090 affects the stability of ChrR. In *E. coli* the proteases DegS and RseP are involved in proteolysis of the RpoE anti-sigma factor RseA. Because the Tn5 mutagenesis did not reveal ^1^O_2_ sensitive protease-mutants in *R. sphaeroides* and the RSP_1090 product has no homology to proteases, the DegS and RseP homologs RSP_3242 and RSP_2710 were deleted in *R. sphaeroides* in order to elucidate if these proteases are involved in ChrR degradation and RpoE activation. Our results support a function of these proteases in singlet oxygen-dependent proteolysis of ChrR. Therefore, central factors involved in RpoE activation are shared between *R. sphaeroides* and *E. coli* despite the limited similarities of the anti-sigma factors ChrR and RseA and the different signals leading to RpoE activation.

## Results

### Insertion of Tn5 in the gene RSP_1090 leads to decreased RpoE activity and increased sensitivity towards ^1^O_2_

**Figure 1:**
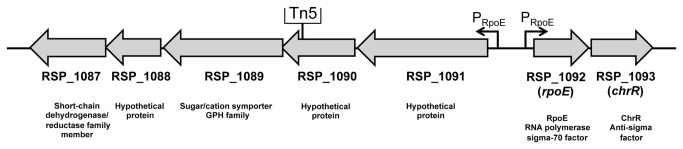
Genetic organization of the RSP_1091-1087 and *rpoEchrR* operons on the *R. sphaeroides* chromosome 1. The insertion site of Tn5 which resulted in reduced RpoE activity is indicated. The Tn5 inserted 683 bp downstream of the start codon of the RSP_1090 gene. RSP_1090 located in a putative operon with RSP_1091, RSP_1089, RSP_1088 and RSP_1087. Both operons are preceded by an RpoE dependent promoter. Annotated protein functions are depicted below the locus tag numbers.

We performed a Tn5 mutagenesis in the *R. sphaeroides* 2.4.1 wild type harboring the reporter plasmid pPHU*phrAlacZ* to identify unknown factors triggering RpoE activation. The plasmid harbors the *lacZ* gene preceded by the RpoE-inducible *phrA* promoter [15]. We screened for those Tn5 mutants which showed decreased or even no β-galactosidase activity upon exposure to ^1^O_2_. Additionally, mutants of interest should not have an insertion of Tn5 in the reporter plasmid pPHU*phrAlacZ* and in the *rpoE* gene including the *rpoE* upstream regulatory region, respectively, and should be more sensitive to ^1^O_2_ than the wild type. Several mutants were found that carried the transposon in the *rpoE* locus and the reporter plasmid, respectively. After screening around 18.000 Tn5 mutants, we finally found one mutant which passed the selection criteria. Vectorette PCR [16] identified the Tn5 insertion into the gene RSP_1090, which encodes a protein of unknown function (Figure 1), that was previously annotated to encode a protein involved in cyclopropane fatty acid synthesis [17]. RSP_1090 is part of the putative RSP_1091-1087 operon located upstream of *rpoEchrR* and belongs to the recently defined RpoE regulon [13,17]. 

### Deletion of RSP_1090 leads to decreased RpoE activity under ^1^O_2_ stress, but not under organic peroxide stress

The deletion of the RSP_1090 gene in the *R. sphaeroides* wild type was performed by the insertion of a kanamycin resistance cassette without transcriptional terminator to avoid polar effects on the transcription of downstream genes. 

**Figure 2:**
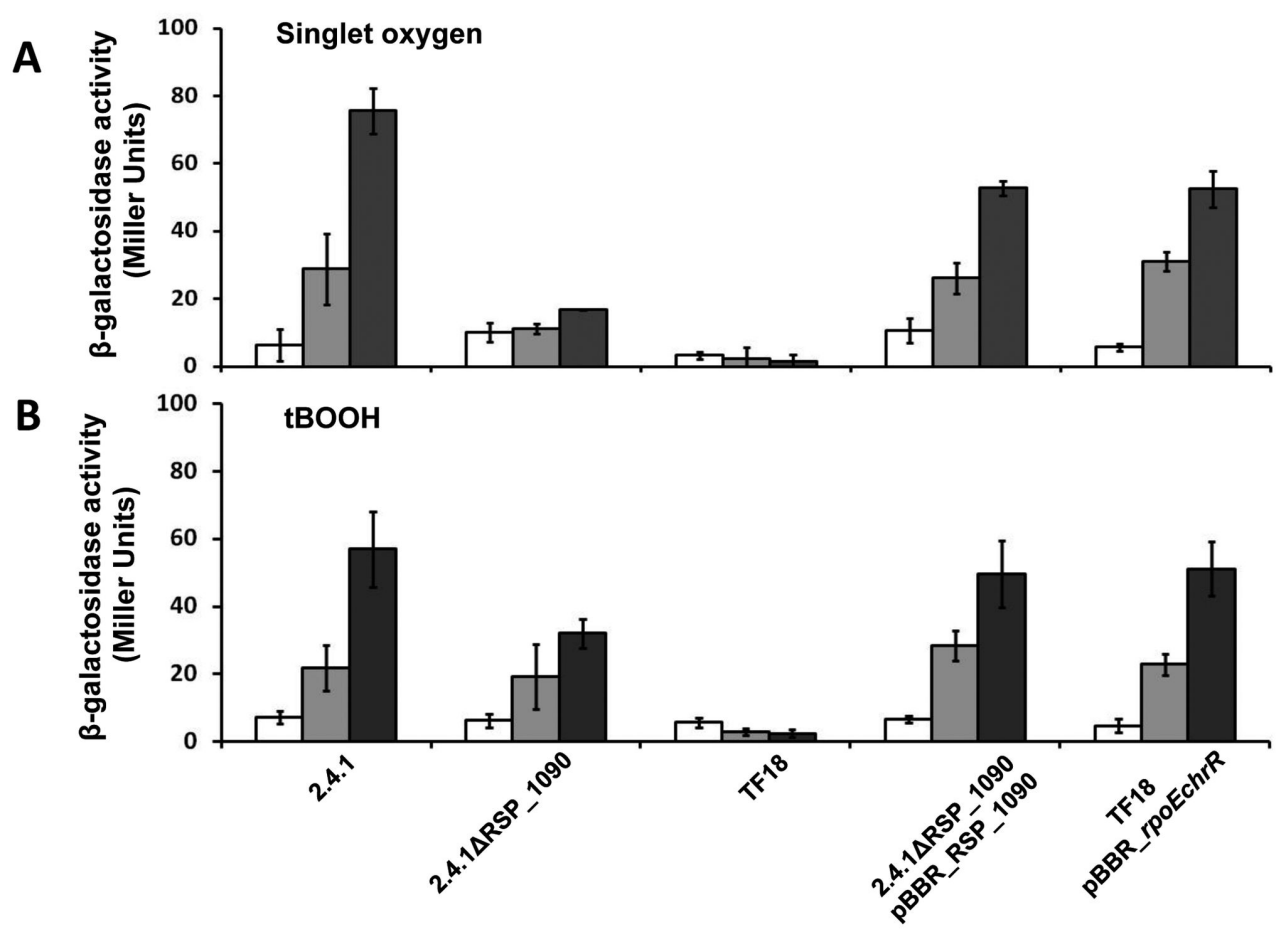
RpoE activity is negatively affected in strain 2.4.1Δ*RSP*_*1090* especially under ^1^O_2_ stress. β-galactosidase activity of the *R. sphaeroides* wild type 2.4.1, strain 2.4.1ΔRSP_1090 and TF18 harboring the reporter plasmid pPHU*phrAlacZ*. Complemented mutant strains were also included. Cells were grown aerobically in the dark to an OD_660nm_ of 0.4 and were exposed to high light (800 W m^-2^) and 50 nM methylene blue (A) or to 360 µM of tBOOH (B) for 0 min, 60 min and 180 min. The data represent the mean of three independent experiments. Error bars indicate the standard deviation.

To analyze the role of RSP_1090 in the RpoE response we monitored RpoE activity via the expression of a *phrA-lacZ* fusion in response to ^1^O_2_ and tBOOH (Figure 2). In the wild type strain β-galactosidase activity increased strongly after ^1^O_2_ exposure (Figure 2A). In contrast to the wild type, a minor increase in β-galactosidase activity was found for 2.4.1ΔRSP_1090. We did not observe any increase in β-galactosidase activity for strain TF18 which lacks *rpoE* and *chrR*. Strain 2.4.1ΔRSP_1090 was complemented with pBBR2.4.1_RSP_1090, harboring RSP_1090 flanked by the RpoE promoter located upstream of the putative RSP_1091-1087 operon. Strain TF18 was complemented with a copy of the *rpoEchrR* operon by using the same vector. Both strains showed higher β-galactosidase activities compared to the deletion strains, but did not match the wild type after 3 h of stress exposure (Figure 2A). For the experiments performed with organic peroxide (Figure 2B), the β-galactosidase activities were similar to those observed for the ^1^O_2_ stress experiment, except for the RSP_1090 deletion strain. RpoE activity was induced in 2.4.1ΔRSP_1090 after organic peroxide exposure, but compared to the wild type the observed activities were significantly lower after 3 h of tBOOH exposure (Figure 2B). The finding that the RpoE activity is strongly impaired in the absence of RSP_1090 under ^1^O_2_ is in agreement with recent studies [14]. 

### Both, RSP_1091 and RSP_1090 are required for full defense against ^1^O_2_

**Figure 3:**
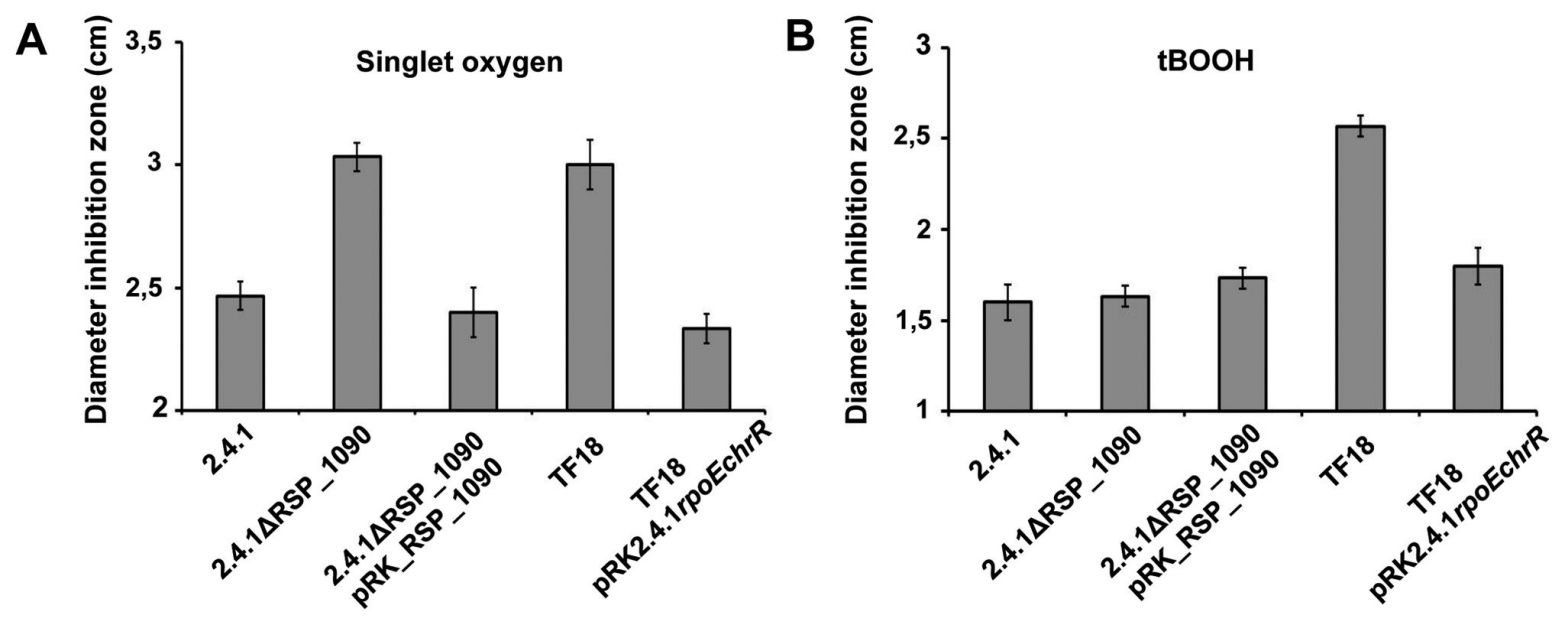
The *RSP_1090* deletion strain is more sensitive to ^1^O_2_ than the wild type. Inhibition of growth of the *R. sphaeroides* wild type 2.4.1, strain 2.4.1Δ1090 and TF18(*rpoEchrR* ^-^) by ^1^O_2_ (A) and organic peroxide (B). The data represent the mean of three independent experiments. Error bars indicate the standard deviation.

The sensitivity of strain 2.4.1ΔRSP_1090 to ^1^O_2_ and organic peroxide was tested by inhibition zone assays (Figure 3). The mutant was more sensitive to ^1^O_2_ compared to the wild type as indicated by larger inhibition zones and similar in sensitivity to the *rpoEchrR* deletion strain TF18 (Figure 3A). In contrast, strain 2.4.1ΔRSP_1090 was as sensitive to organic peroxide as the wild type, but strain TF18 showed a higher sensitivity (Figure 3B). 

Both complemented mutant strains, 2.4.1ΔRSP_1090pRK_RSP_1090 and TF18pRK2.4.1*rpoEchrR*, showed a similar sensitivity to ^1^O_2_ and to organic peroxide as the wild type (Figure 3 A and B). We also tested the sensitivity of the strains 2.4.1ΔRSP_1090, TF18 and the wild type harboring the empty vector pRK415 and we did not observe any difference in sensitivity compared to the respective strains lacking pRK415 (Table S1). 

**Figure 4:**
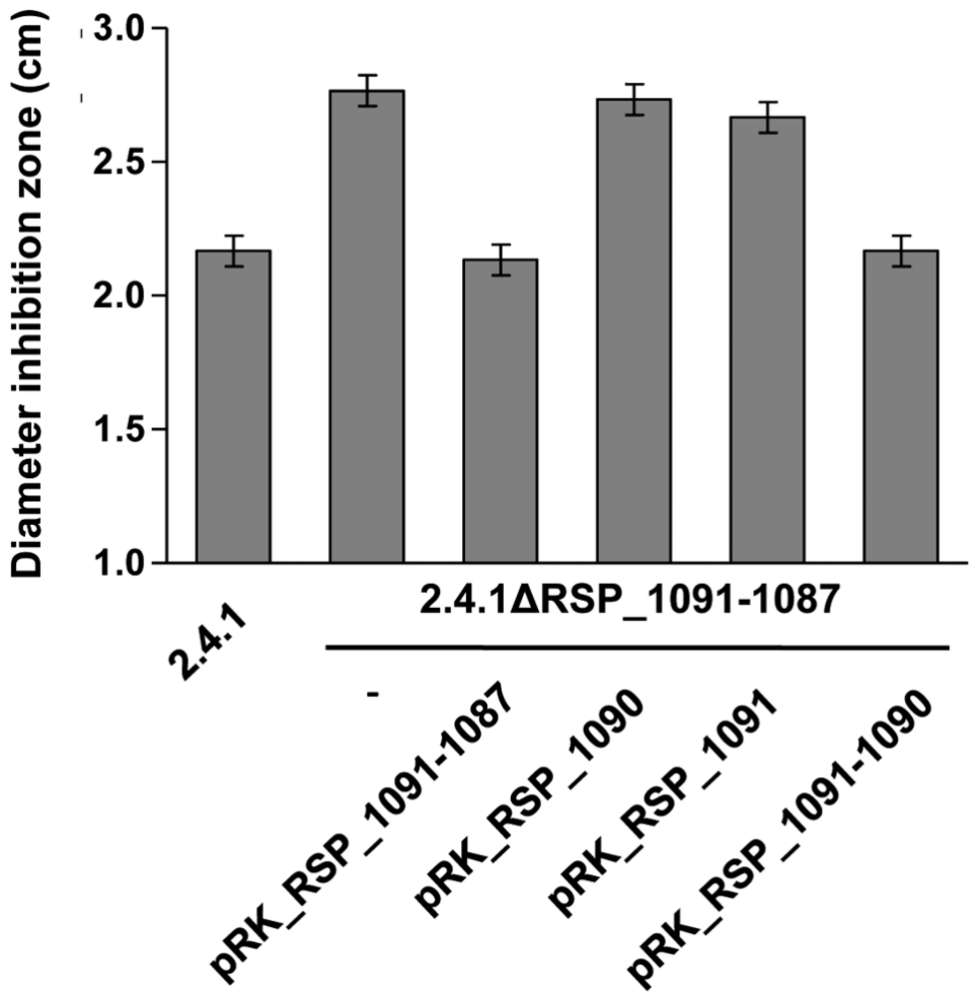
Inhibition by ^1^O_2_ in 2.4.1ΔRSP_1091-1087 complementation strains. Inhibition of growth by ^1^O_2_ of the *R. sphaeroides* wild type 2.4.1, strain 2.4.1ΔRSP_1091-1087 and complementation with RSP_1091-1087, RSP_1090, RSP1091 and RSP_1091-1090 in pRK415. The data represent the mean of three independent experiments. Error bars indicate the standard deviation.

To address the question which genes of the RSP_1091-1087 operon are required for full activation of RpoE and to exclude polar effects of the RSP_1090 deletion strain, we deleted the entire RSP_1091-1087 operon in the *R. sphaeroides* wild type by the insertion of a kanamycin resistance cassette. Complementation of the mutant was then performed by reintroducing either the entire operon, RSP_1091, RSP1090 or a combination of RSP1091 and RSP1090 in trans on pRK415. Significantly larger inhibition zone assays showed that the RSP1091-1087 mutant was more sensitive to ^1^O_2_ compared to the wild type (Figure 4). Reintroduction of RSP_1091-1087 on pRK415 fully restored the wild type phenotype. It was not possible to restore the wild type phenotype by reintroducing either RSP1090 or RSP1091 on a low copy plasmid, because inhibition zones were similar to those observed for strain 2.4.1ΔRSP_1091-1087 (Figure 4). Only a combination of both genes restored the wild type phenotype. Therefore, RSP1091 and RSP1090 are both required for defense against ^1^O_2_ stress and full activation of the RpoE-dependent ^1^O_2_ stress response. 

### The RSP_1091-1090 locus is well conserved in the α-proteobacteria

Homologs of RSP_1090 proteins were identified by using the BLAST option on the integrated microbial genome (IMG) website. In the Bacteria a number of 337 genomes contained RSP_1090 with an upstream encoded homolog of RSP_1091, in most cases in proximity to *rpoE* and *chrR* homologs. RSP_1090 and RSP1091 were annotated in *R. sphaeroides* to be related to putative cylcopropane/cyclopropene fatty acid synthesis proteins. However, this annotation appears not to be justified due to the weak homologies to verified cylcopropane/cyclopropene fatty acid synthesis proteins. RSP_1091 and RSP_1090 were found in many α-proteobacteria, whereas conservation of RSP_1089, RSP_1088 and RSP_1087 is restricted to species belonging to the *Rhodobacteraceae* as e.g. *Roseobacter denitrificans* OCh114 (Figure S1). However, the RSP_1089-1087 homologs are absent in more distantly related *Rhodobacteraceae* as *Oceanicola granulosus* HTCC2516 (Figure S1), which underlines a genetic context specific to bacteria closely related to *Rhodobacter*. Also *Caulobacter* *sp.* K31 harbors RSP_1091 and RSP_1090 homologs encoded in close distance to *rpoE* and *chrR* homologs (Figure S1), but in other *C. crescentus* strains those homologs were not located together with *rpoE* and *chrR* homologs. In *Rhizobium etli* CIAT 652 no RSP_1091 and RSP_1090 homologs were located close to a *chrR* homolog, but *rpoE* was missing (Figure S1). In summary, the RSP_1091 and RSP_1090 homologs are well conserved in the α-proteobacteria and are frequently encoded adjacent to *rpoE* and *chrR* homologous genes.

### The ChrR protein is rapidly degraded under singlet oxygen stress in the presence of RSP_1090

**Figure 5:**
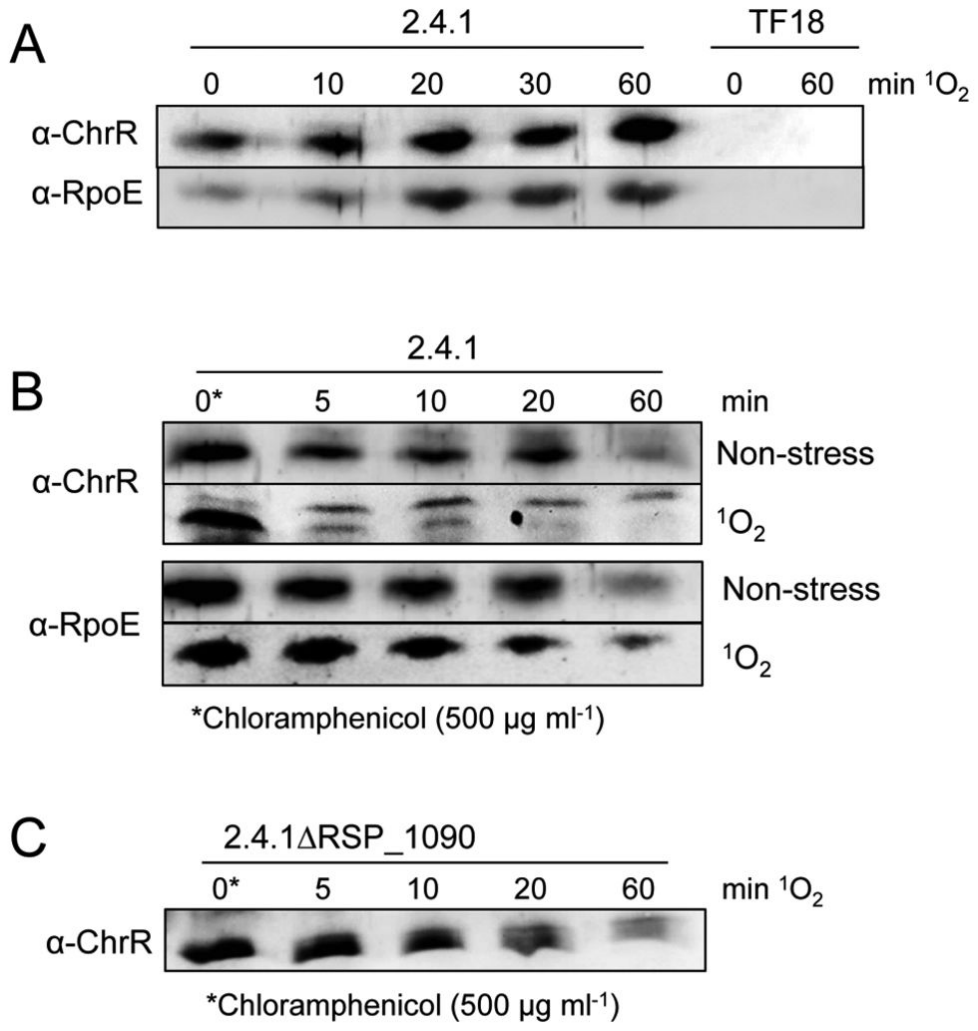
Protein levels and stabilities of RpoE and ChrR under non-stress and ^1^O_2_ stress conditions. For Western blotting 240 µg of total protein were used. Loading of equal amounts of proteins was confirmed by Ponceau staining (not shown). Antibodies (α-RpoE and α-ChrR) were raised against the recombinant His_6_-tagged RpoE and ChrR proteins, respectively. ^1^O_2_ stress was induced at time point 0 min (OD_660nm_ 0.4). (A) Levels of RpoE and ChrR in the *R. sphaeroides* wild type and the TF18 strain at different time points of ^1^O_2_ exposure (high light 800 W m^-2^; 50 nM methylene blue). (B) Stability of RpoE and ChrR in the *R. sphaeroides* wild type under non-stress (50 nM methylene blue; dark) and ^1^O_2_ stress conditions (high light 800 W m^-2^; 50 nM methylene blue). (C) Stability of ChrR in the presence of ^1^O_2_ in the RSP_1090 deletion mutant. To check ChrR stability under stress conditions, translation was inhibited by adding chloramphenicol (500 µg ml^-1^) after cultures were exposed for 60 min to ^1^O_2_ (time point 0 min). For non-stress conditions chloramphenicol was added 1 hour after OD_660nm_ 0.4 (time point 0 min), while cultures were further incubated in the dark under aerobic conditions. The wild type control is depicted in Figure 5B. Western blots were developed using α-RpoE and α-ChrR, respectively, and anti-rabbit IgG conjugated with alkaline phosphatase.

For *R. sphaeroides* we analyzed the levels of ChrR and RpoE in the *R. sphaeroides* wild type under ^1^O_2_ stress and non-stress conditions (Figure 5A). Polyclonal antibodies raised against the His_6_-tagged version of ChrR and RpoE were applied to detect changes in the levels of both proteins. ChrR and RpoE were detected in the absence of ^1^O_2_ (0 min, Figure 5A) and the level of both proteins increased within 60 min of ^1^O_2_ exposure. In strain TF18, neither protein was detectable (Figure 5A). Because proteolysis of the cognate anti-sigma factor is one known mechanism for sigma factor activation in Gram negative and Gram positive bacteria [18], we tested the stability of ChrR and RpoE after translation inhibition using chloramphenicol (Figure 5B). If RpoE activity is regulated by ChrR proteolysis, ChrR stability should be negatively affected under ^1^O_2_ stress conditions. In the presence of ^1^O_2_ and chloramphenicol two bands specific for ChrR were detected (Figure 5B). Without exposure to ^1^O_2_ ChrR was stable for at least 20 min in the *R. sphaeroides* wild type, but only a faint signal was detected after 60 min. In the presence of ^1^O_2_, the signal for the lower ChrR band was strongly decreased within 5 min, but the upper band was detectable even after 60 min. In contrast, RpoE was rather stable for 20 min under both conditions and detected in lower amounts after 60 min of ^1^O_2_ stress conditions (Figure 5B). As RpoE activity and resistance to ^1^O_2_ are decreased in the absence of RSP_1090, we tested for ChrR proteolysis in the RSP_1090 deletion strain. ChrR was more stable in the RSP_1090 mutant under ^1^O_2_ stress conditions (Figure 5C) compared to the wild type strain (Figure 5B). Its half-life in the RSP_1090 deletion strain was similar to the wild type under non-stress conditions (Figure 5B and C). Our results therefore verify that ChrR stability is decreased upon exposure of *Rhodobacter* to ^1^O_2_ stress and that degradation of ChrR demands RSP_1090 [13,14].

### RSP_1090 dependent ChrR degradation does not require de novo synthesis of proteases

**Figure 6:**
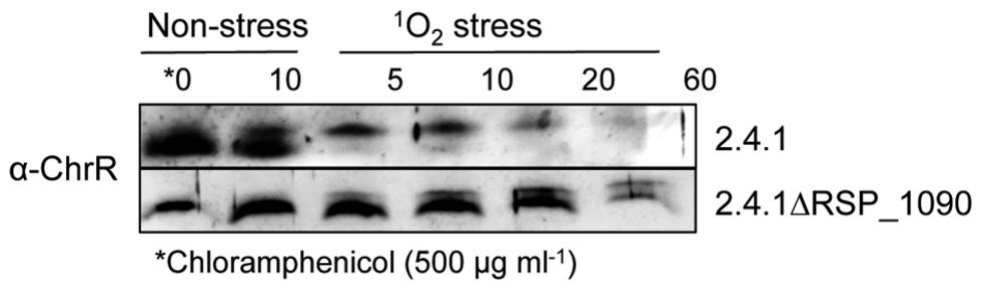
Protein stabilities of ChrR in the *R. sphaeroides* wild type 2.4.1 and strain *2.4.1ΔRSP_1090*. For Western blotting 240 µg of total protein were used. Loading of equal amounts of proteins was confirmed by Ponceau staining (not shown). Stability of ChrR in the wild type and strain 2.4.1ΔRSP_1090, with chloramphenicol treatment 10 min before induction of ^1^O_2_ stress (high light 800 W m^-2^; 50 nM methylene blue).

ChrR is rapidly degraded under ^1^O_2_ exposure, but it remained unclear if the involved protease/proteases are already synthesized prior to stress exposure and therefore proteolytic activity is increased upon stress exposure or if *de novo* synthesis of involved factors is required. To address this question, we analyzed ChrR stability in the wild type strain by adding chloramphenicol 10 minutes before stress induction (Figure 6). ChrR levels were similar before and 10 min after addition of chloramphenicol in the absence of ^1^O_2_. ChrR was then rapidly degraded under ^1^O_2_ exposure and the signal of the lower ChrR band was abolished after 5 min of stress exposure (Figure 6). Required factors leading to ChrR degradation are therefore present before stress exposure and not synthesized *de novo*. To test for contribution of RSP_1090 for the activation of ChrR proteolysis, we repeated the above described experiment with the RSP1090 mutant. In contrast to the wild type, ChrR was detectable in 2.4.1∆RSP_1090 (Figure 6) even after 60 min of stress. The results indicate that the respective protease/proteases involved in ChrR proteolysis are present under non-stress conditions and that ChrR proteolysis is activated upon ^1^O_2_ stress exposure. This process depends on RSP1090, which as well does not require *de novo* protein synthesis. 

### DegS and RseA type proteases are involved in RpoE activation in *R. sphaeroides*

In *E. coli* RpoE is involved in regulation of the membrane stress response [9]. Its activity is controlled by the membrane bound anti-sigma factor RseA, which undergoes regulated proteolysis. First the trypsin-like serine endoprotease DegS cleaves the periplasmic domain of RseA, then the transmembrane domain is cleaved by the zinc-metallo protease RseP. Finally cytoplasmic proteases degrade the part of RseA, which is still bound to RpoE [19] and RpoE can consequently activate its target genes. The *R. sphaeroides* protein RSP_3242 shares 37 % identity with the *E. coli* DegS protein and the RSP_2710 protein shares 31 % identity with RseP. To test whether these proteases have a similar function in RpoE-dependent signaling in *R. sphaeroides* as in *E. coli* we constructed strains lacking the respective genes. In addition we constructed a strain lacking the RSP_1096/1097 genes, which are in close neighborhood to the *rpoE-chrR* operon on the chromosome and encode a putative zinc-metallo protease. All strains showed similar growth behavior as the wild type (data not shown).

**Figure 7:**
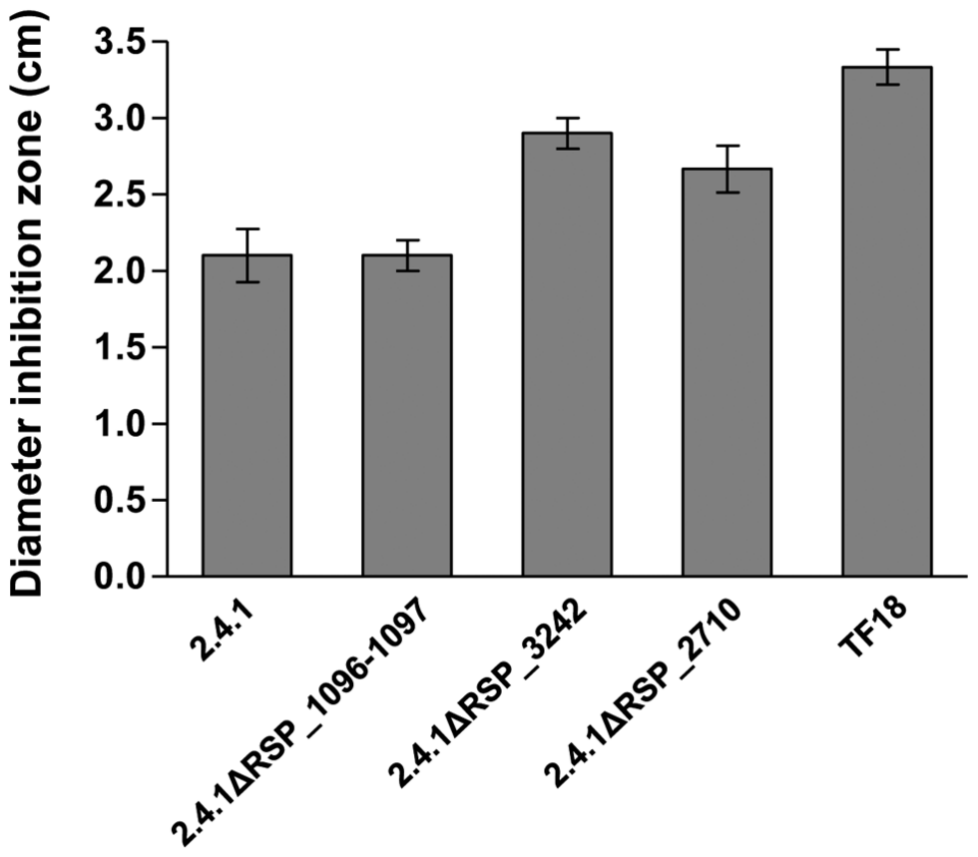
Deletion of the *degS* and *rseP* homologous genes RSP_3242 and RSP_2710 affects sensitivity to ^1^O_2_. Inhibition of growth of the *R. sphaeroides* wild type 2.4.1, strains 2.4.1ΔRSP_1096/1097, 2.4.1ΔRSP_3242, 2.4.1ΔRSP_2710 and TF18(*rpoEchrR* ^-^) by ^1^O_2_. The data represent the mean of three independent experiments. Error bars indicate the standard deviation.

Less efficient proteolytic degradation of ChrR should result in lower activity of RpoE and consequently in lower resistance to ^1^O_2_. Therefore we tested the sensitivity of all three mutants against this substance. Strain 2.4.1ΔRSP_1096-1097 showed similar sensitivity in inhibition zone assays as the parental wild type strain, indicating no major role of the deleted genes in RpoE signaling (Figure 7). In contrast, the strains 2.4.1ΔRSP_3242 and 2.4.1ΔRSP_2710 showed significantly increased sensitivity against ^1^O_2_ when compared to the wild type. Their sensitivity was, however, lower than that of strain TF18. 

**Figure 8:**
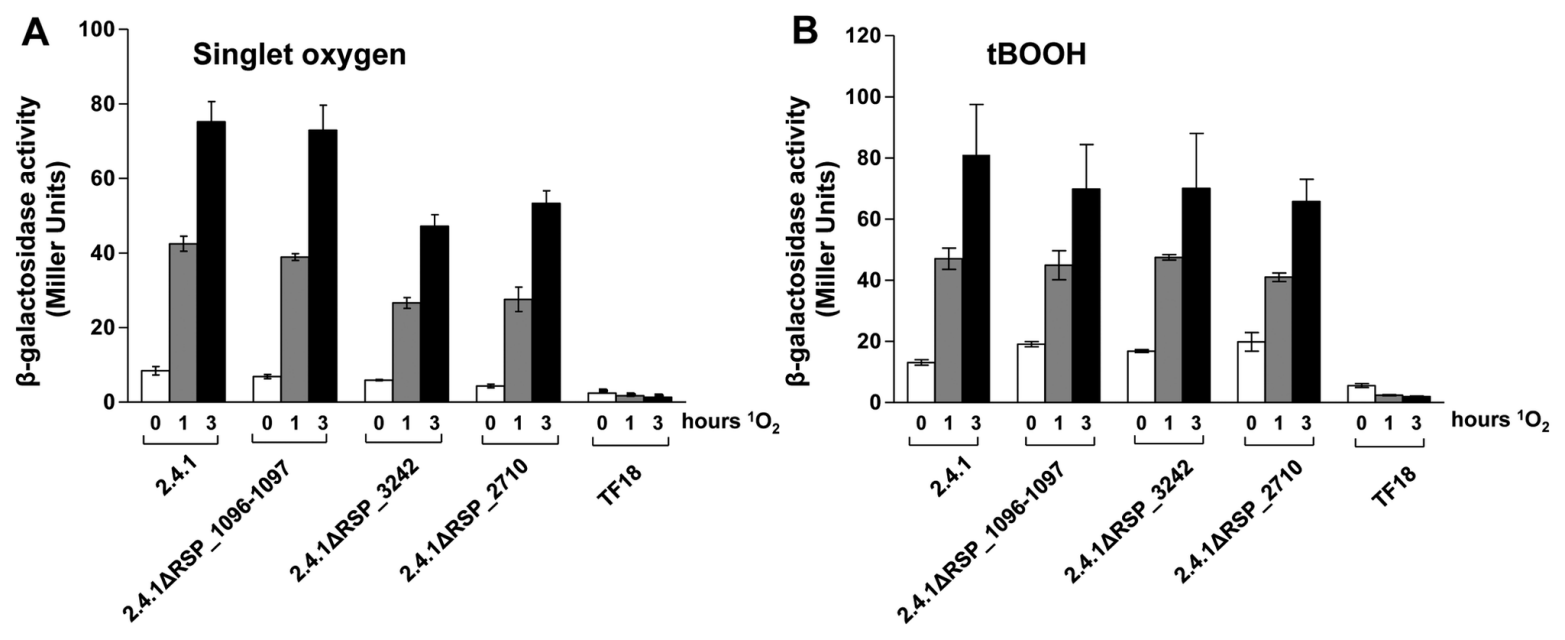
RpoE activity is negatively affected in strains 2.4.1ΔRSP_3242 and 2.4.1ΔRSP_2710. β-galactosidase activity of the *R. sphaeroides* wild type 2.4.1 and strains 2.4.1ΔRSP_1096/1097, 2.4.1ΔRSP_3242, 2.4.1ΔRSP_2710 and TF18 harboring the reporter plasmid pPHU*phrAlacZ*. Cells were grown aerobically in the dark to an OD_660nm_ of 0.4 and were exposed to high light (800 W m^-2^) and 50 nM methylene blue (A) or to 360 µM tBOOH (B). The data represent the mean of three independent experiments. Error bars indicate the standard deviation.

To further elucidate the role of the three proteases in RpoE-dependent signaling the expression of an *phrA-lacZ* reporter gene in response to ^1^O_2_ and organic peroxide was analyzed. While the increase of β-galactosidase activity after the ^1^O_2_ exposure was nearly identical in the wild type and strain 2.4.1ΔRSP_1096-1097, the increase of β-galactosidase activity was clearly reduced in strains 2.4.1ΔRSP_3242 and 2.4.1ΔRSP_2710 (Figure 8A). As a negative control strain TF18 was used, which is lacking *rpoEchrR* and therefore exhibits no or only basal β-galactosidase activity. In contrast to ^1^O_2_, organic peroxide exposure did not lead to significantly decreased expression of the *phrA-lacZ* reporter gene in the protease mutant strains (Figure 8B).

Our results suggest a role of RSP_3242 and RSP_2710 in ChrR degradation. Therefore we directly tested the turn-over of ChrR in these two mutant strains. 

### DegS and RseA type proteases are involved in ChrR degradation in *R. sphaeroides*

We compared the decay of ChrR in the presence of ^1^O_2_ in the mutants lacking RSP_3242 or RSP_2710 to the decay in wild type cells. In all strains we observed two ChrR specific bands. While the lower band was maximal before addition of chloramphenicol and showed decreased abundance after its addition, the upper band strongly increased directly after addition of chloramphenicol and disappeared at later time points. 

**Figure 9:**
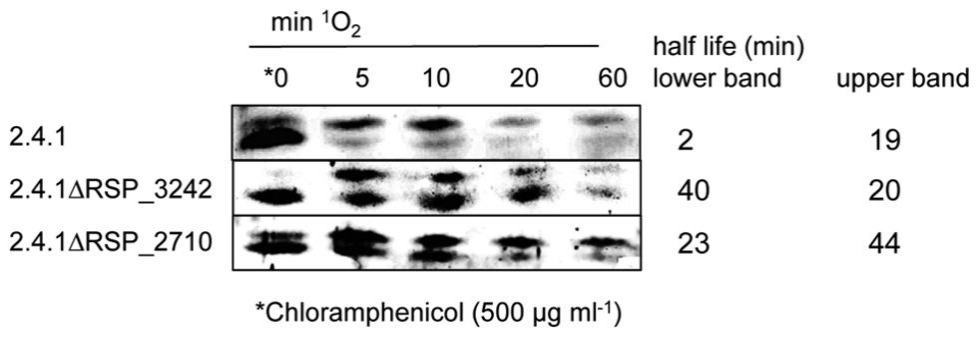
Deletion of *DegS* and *RseA* like proteases increases ChrR stability. Stability of ChrR in the *R. sphaeroides* wild type and strains 2.4.1ΔRSP_3242, 2.4.1ΔRSP_2710 under ^1^O_2_ stress conditions (high light 800 W m^-2^; 50 nM methylene blue). To check ChrR stability under stress conditions, translation was inhibited by adding chloramphenicol (500 µg ml^-1^) after cultures were exposed for 60 min to ^1^O_2_ (time point 0 min). Western blots were developed using α-ChrR and anti-rabbit IgG conjugated with alkaline phosphatase.

The half-life of the lower band clearly increased in the RSP_3242 and RSP_2710 mutants compared to the wild type (Figure 9). In the wild type strain the upper ChrR band had a much longer half-life than the lower band. The half-life of the upper band strongly increased in the RSP_2710 mutant. These data support a contribution of the DegS and RseA homologs in proteolytic degradation of the ChrR anti sigma factor. 

## Discussion

### RpoE activation under ^1^O_2_ stress is regulated by proteolysis of ChrR and requires RSP_1090 and RSP1091

In this study we demonstrate that under ^1^O_2_ stress RpoE is activated by rapid proteolysis of the anti-sigma factor ChrR and show that ChrR proteolysis under ^1^O_2_ stress is dependent on RSP_1090. Our result is in line with the recent finding that an in frame deletion of RSP_1091-1090 leads to decreased activation of *rpoE* expression in the presence of ^1^O_2_ and that ChrR proteolysis demands RSP_1090 [13,14]. Nam et al. [14] reported that isolation of an in frame deletion of only RSP_1090 was not possible and suggested that the corresponding protein may be needed for viability in the absence of ^1^O_2_. The isolation of an RSP_1090 Tn5 mutant and construction of a knock out of RSP_1090 in this study demonstrate that the gene is not essential and failure to obtain a mutant may rather be due to technical reasons. The finding that the RSP_1090 deletion strain is negatively impaired in response to ^1^O_2_, but not to organic peroxide, strongly indicates that ^1^O_2_ and organic peroxide act independently on RpoE activation. 

Moreover, we demonstrate that RSP_1090 triggered ChrR proteolysis under ^1^O_2_ does not require *de novo* synthesis of proteases. The required proteases are already present before stress exposure and proteolysis of ChrR is quickly activated upon stress exposure. This allows a rapid activation of the RpoE response and subsequent stress adaptation. We demonstrate that both genes RSP_1091 and RSP_1090 are required for full resistance to ^1^O_2_ and that RSP_1091-1090 homologs and their genetic location are highly conserved in other genomes. This finding supports that a combination of both genes is required for full activation of RpoE dependent defense mechanisms. As in *R. sphaeroides*, in many other genomes the RSP_1090 and RSP_1091 homologs are encoded next to sigma factor encoding genes, often co-localized with an anti-sigma factor encoding gene. The activity of those sigma factors may be controlled as well by RSP_1090 and RSP_1091 dependent proteolysis of the cognate anti-sigma factor, which most likely represents a highly conserved mechanism. 

### Organic peroxide stress activates RpoE via ChrR proteolysis, but does not depend on RSP_1091 and RSP_1090

Exposure of *R. sphaeroides* to organic peroxide leads to RpoE activation and ChrR proteolysis [14]. Here we further demonstrate that RpoE activation in the presence of organic peroxide does not require RSP_1090 or the DegS and RseP homologs, indicating that the response to ^1^O_2_ and organic peroxide is mediated via different signal chains to RpoE. It was recently shown that organic peroxide and ^1^O_2_ promote the dissociation of the RpoE:ChrR complex and that dissociation involves the ChrR C-terminal domain, which contains two conserved cysteine residues [20]. Oxidants such as organic peroxide are known to affect proteins via e.g. the modification of cysteine residues [21]. Organic peroxide might promote RpoE:ChrR dissociation by oxidation of one or both of the two cysteines within the ChrR C-terminal domain, as hypothesized recently [14]. Free ChrR could be targeted by proteases different from DegS and RseP. How organic peroxide eventually leads to RpoE activation and the identification of the involved protease remains to be elucidated.

### Activation of the RpoE response in *R. sphaeroides* shows homology to the RpoE/RseA system in *E. coli*

As RSP_1090 and RSP_1091 do not show any homology to known proteases, we assume that proteolysis is not directly linked to the RSP_1090-1091 gene products. In *E. coli* RpoE is activated upon cell envelope stress by the stepwise proteolysis of the cognate anti-sigma factor RseA, due to the proteolytic activity of DegS and RseP [9]. In *R. sphaeroides* homologs to DegS and RseP exist. As the anti-sigma factor domains (ASD) of RseA and ChrR exhibit structure homology [7], the DegS and RseP homologs RSP_3242 and RSP_2710 were possible candidates for ChrR proteolysis in *R. sphaeroides*. In fact, our results show the involvement of RSP_3242 and RSP_2710 in ChrR proteolysis upon ^1^O_2_ stress exposure. Sigma factor activation by proteolysis of the cognate anti-sigma factor is a common mechanism within bacterial species [18] and was recently shown for RpoE/ChrR [14].

Besides RseA, RpoE activity in *E. coli* is negatively regulated by RseB, a protein which directly binds to the periplasmic domain of RseA [22-24]. Binding of RseB to RseA prevents RseP from degrading intact RseA, ensuring that RseA proteolysis is only initiated when DegS is activated upon stress [24]. A further signal is required that inhibits RseB, as RseB binding to RseA prevents cleavage by activated DegS [9,25,26]. A recent study provides evidence that intermediates in LPS transport and assembly are the second signal for RpoE activation, in this context LPS antagonizes RseA-RseB binding [27]. 

A RseB homolog was not found in the *R. sphaeroides* genome, but an RseB like action of RSP_1091 and RSP_1090 in *R. sphaeroides* is conceivable. RSP_1091 exhibits a putative transmembrane domain and could therefore be membrane-localized. Similar to RseA, ChrR could be membrane-localized as it exhibits a putative N-terminal transmembrane domain [28]. Membrane localization of the RpoE:ChrR complex is supported by Western blot experiments in which RpoE and ChrR were both detected in soluble (periplasmic/cytoplasmic) and insoluble (membrane) protein fractions. In non-stressed and ^1^O_2_ stressed wild type cultures RpoE and ChrR were more abundant in the insoluble fractions (data not shown). According to DegS and RseP, the proteases RSP_3242 and RSP_2710 carry at least one putative membrane-spanning segment. The subcellular localization of RSP_1091, RSP_1090 and ChrR and a possible interaction between these proteins will be investigated in future studies.

Interestingly, RSP_1091 is predicted to bind FAD or NAD. The N-terminus of the *R. sphaeroides* AppA protein was found to bind FAD non-covalently and was later termed BLUF (blue light using FAD). The BLUF domain was shown to function as a novel photoreceptor [29,30]. RSP_1091 could be involved in light- or redox-dependent sensing of ^1^O_2_ and might transmit the signal to RSP_1090, RSP_3242 or RSP_2710 to trigger ChrR proteolysis. The light- or redox- dependent activation of RpoE would be one of at least two possible mechanisms of RpoE activation, as RpoE activation by organic peroxide is light independent. Further studies on the localization of the involved factors and the function of RSP_1091 and RSP_1090 are in progress to unravel the detailed mechanism of ^1^O_2_ dependent RpoE activation.

### Singlet oxygen signal transduction in *R. sphaeroides*

How ^1^O_2_ is sensed and recognized by the cells is far from being unraveled, but this study provides important insights into the conversion of the ^1^O_2_ signal to a transcriptional response in *R. sphaeroides*. In the previous model of RpoE activation [13] proteases were not included. Our results and another recent study [14] provide the experimental evidence that RSP_1091 and RSP_1090 are required for RpoE activation. It is important to note that the RSP_1091-1087 operon is under RpoE control [4] and induced by ^1^O_2_ exposure [31], suggesting a regulatory feedback loop. RSP_1091 and RSP_1090 are expressed under non-stress conditions, but the mRNA levels increase at least 4 fold after ^1^O_2_ exposure [31]. Once RpoE is activated upon stress induction, the expression of the *rpoEchrR* operon itself and the RSP_1091-1087 operon is induced by RpoE. When we analyzed RpoE and ChrR protein levels and stabilities under ^1^O_2_ stress (Figure 5 A and B), we observed increasing RpoE and ChrR levels over time in equal amounts, but interestingly ChrR stability was highly decreased under ^1^O_2_ stress in contrast to RpoE stability. This finding indicates that the ChrR turnover rate is much higher under ^1^O_2_ stress compared to RpoE. Therefore, RSP_1091 and RSP_1090 seem to be crucial to further enhance ChrR proteolysis to keep RpoE activity high during stress exposure. 

The proteases RSP_2710 and RSP_3242, which are involved in proteolytic degradation of ChrR are not induced by ^1^O_2_ and not controlled by RpoE [31]. Envelope stress in *E. coli* that leads to dissociation of the anti-sigma factor RseA from RpoE is initiated by misfolding and assembly of outer membrane proteins in the periplasm [32]. In detail, the DegS protease is activated by unassembled porin monomers [10]. Several periplasmic or membrane stress factors lead to activation of DegS and subsequent release of RpoE [9]. Therefore, the mechanism of converting a stress signal as ^1^O_2_ formation into a cascade that leads to activation of RpoE may be highly similar in *R. sphaeroides* compared to what is known in *E. coli*, despite the cognate anti-sigma factors are not homologous. 

## Conclusions

**Figure 10:**
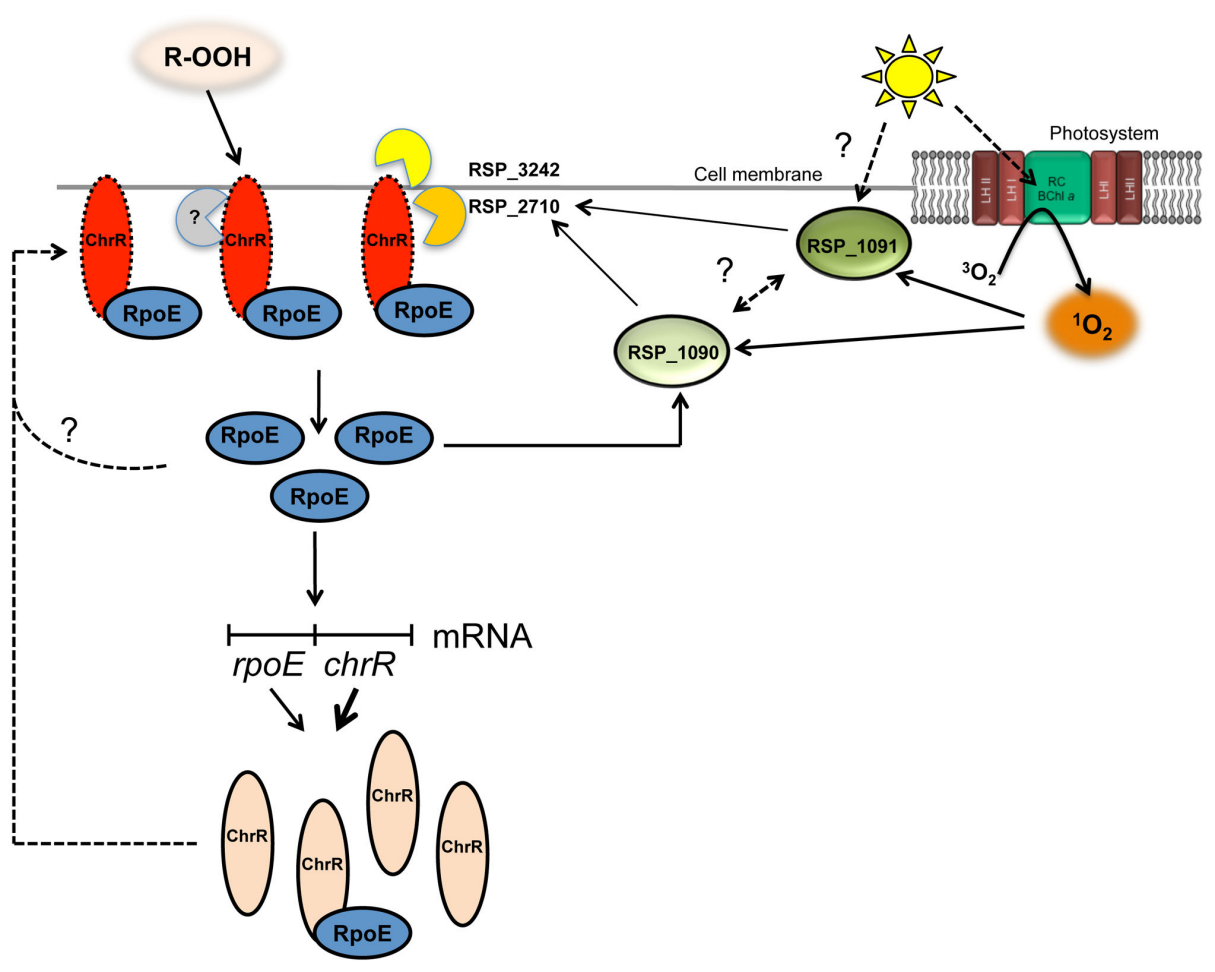
Current model of RpoE activation by ChrR proteolysis in *R. sphaeroides* under ^1^O_2_ stress. The model displays the mechanism of RpoE activation in the response to ^1^O_2_. The localization of ChrR at the membrane is speculative. Solid black arrows indicate positive effects. Dashed arrows indicate hypothetical effects. A detailed explanation is given in the conclusion part.

Our current model (Figure 10) shows the activation of RpoE in the response to photooxidative stress. Under non-stress conditions the ChrR proteolysis rate is low, but ensures a basal level and activity of RpoE in the cell. Upon exposure to ^1^O_2_, as well as organic peroxide stress, the anti-sigma factor ChrR is rapidly degraded. The ^1^O_2_ induced proteolysis requires RSP_1091 and RSP_1090 and involves at least two proteases, the DegS and RseP homologs RSP_3242 and RSP_2710. ChrR proteolysis leads to RpoE release, RpoE binds to the RNA-polymerase and induces target gene expression, including the *rpoEchrR* and the RSP_1091-1087 operon. Increased levels of RSP_1091 and RSP_1090 promote ongoing ChrR proteolysis to maintain high RpoE activity, which displays a positive regulatory loop. Activation of RpoE in the presence of organic peroxide does not require the DegS and RseP homologs RSP_3242 and RSP_2710 for ChrR proteolysis, but so far unknown proteases. In this study further components of the cascade involved in ^1^O_2_ signaling were identified, but the direct link between RSP_1091 and RSP_1090 and the proteases RSP_2710 and RSP_3242 in ChrR proteolysis and subsequent RpoE activation remain to be elucidated. 

## Material and Methods

### Bacterial strains and growth conditions

*R. sphaeroides* strains were grown at 32°C in minimal salt medium containing malate as carbon source [33]. Aerobic growth conditions with a concentration of 160 to 180 µM of dissolved oxygen were established by gassing cultures with air in flat glass bottles or by continuous shaking of Erlenmeyer flasks at 140 rpm with a culture volume of 20%. In semiaerobic cultures a volume of 80% in Erlenmeyer flasks and shaking at 140 rpm lead to a dissolved oxygen concentration of approximately 25 µM. When necessary kanamycin (25 µg ml^-1^), tetracycline (1.5 µg ml^-1^), trimethoprim (50 µg ml^-1^) or gentamycine (10 µg ml^-1^) was added to liquid and solid growth media (1.6% agar). Antibiotics were omitted from pre-cultures, cultures and agar plates used for *R. sphaeroides* strains during stress experiments and inhibition zone assays (see below). *E. coli* strains were grown aerobically at 37°C in LB medium under continuous shaking at 180 rpm or on solid growth media.

### Sensitivity to ^1^O_2_ and organic peroxide

Measurement of sensitivity to ^1^O_2_ was performed as described before [34]. The measurement of sensitivity to organic peroxide was performed similarly to ^1^O_2_ experiments. Instead of methylene blue 5 µl of 700 mM tBOOH were added to the filter disks and the agar plates were incubated 48 hours in the dark.

### Tn5-mutagenesis of *R. sphaeroides* 2.4.1pPHU*phrAlacZ*

The *R. sphaeroides* wild type harboring plasmid pPHU*phrAlacZ* was grown under semiaerobic conditions in the presence of 1.5 µg ml^-1^ tetracycline. The *E. coli* strain S17-1pSUP202 was grown aerobically in LB medium containing 20 µg ml^-1^ tetracycline. For Tn5-mutagenesis 1 ml of exponential-phase *R. sphaeroides* culture was mixed with 200 µl exponential-phase *E. coli* culture. The cells were centrifuged at 5.000 rpm for 5 min at room temperature. The supernatant was removed and the cells were washed in 1 ml of malate minimal medium. After a second centrifugation the supernatant was discarded and the cells resuspended in 200 µl of malate minimal medium. The cell suspension was transferred onto nitrocellulose membranes (Whatman, Dassel, Germany) placed on PY agar plates. After 5 hours of incubation at 32°C the filter was transferred into a fresh 1.5 ml tube containing 1 ml of malate minimal medium. After vortexing, the whole suspension was diluted in malate minimal medium and 50 µl aliquots were plated on malate minimal agar plates containing 2 µM of Rose Bengal, tetracycline (1.5 µg ml^-1^) and kanamycin (25 µg ml^-1^). The plates were incubated at 32°C for 3 days in the dark.

### Screening for Tn5-mutants with decreased β-galactosidase activity under ^1^O_2_ stress

Agar plates containing *R. sphaeroides* colonies were placed under a daylight fluorescent tube (20 W m^-2^) to induce RpoE activity. After 2 hours the plates were sprayed with X-Gal (20 mg ml^-1^) and incubated for 6 hours at room temperature. Such colonies were further investigated, which were not or only slightly colored blue. To test for Tn5 insertion into plasmid pPHU*phrAlacZ*, and consequently leading to a false negative result, pPHU*phrAlacZ* was isolated from Tn5 mutants and electroporated into *E. coli* JM109. The *E. coli* cells were plated on LB agar plates containing 20 µg ml^-1^ tetracycline (pPHU*phrAlacZ*) and 25µg ml^-1^ kanamycin (Tn5). Cells growing on kanamycin had a Tn5 insertion in the plasmid pPHU*phrAlacZ* and respective Tn5 mutants were excluded. 

### Identification of Tn5 insertion sites by vectorette PCR

In principle, the Vectorette PCR was performed as described previously [16]. For the synthesis of the Vectorette units two imperfect complementary DNA oligonucleotides (Vectorette oligonucleotide_1 and Vectorette oligonucleotide_2, Table S2) were incubated for 5 min at 65°C. After addition of 5 µl 25 mM MgCl_2_ solution the oligonucleotides were cooled down slowly to room temperature. After digestion of chromosomal DNA with the blunt end restriction enzymes PvuII, FspI and DpnI (New England Biolabs), the digested DNA was purified with phenol/chloroform/isoamylalcohol (AppliChem, Darmstadt, Germany) and precipitated with ethanol and sodium acetate. The Vectorette units were ligated with the digested DNA, ligation was performed for 12 hours at 15°C. The ligated DNA was used as template in a PCR reaction using Taq DNA polymerase (Qiagen) and the primers 224new and Tnp_out_new (Table S2). After PCR the samples were separated on a 1% agarose gel and DNA fragments were gel extracted with the Gel extraction kit QIAEX III (Qiagen). The purified DNA fragment was cloned into the pDrive vector (Qiagen) and sequenced with the primer Tnp_out_new. 

### β-galactosidase activity assay

This was carried out as described previously [35].

### Photooxidative stress conditions

Photooxidative stress conditions were performed as described earlier [5], except the final concentration of methylene blue. In brief, cultures were grown under semiaerobic conditions over night to obtain pigmented cells. Cultures were diluted to an OD_660nm_ of 0.2 and allowed to double once under aerobic growth conditions in darkened flat glass bottles. High light conditions were generated by illumination with 800 W m^-2^ white light. For photooxidative stress ^1^O_2_ producing methylene blue was added to liquid cultures at a final concentration of 50 nM prior to illumination. 

### Construction of *R. sphaeroides* deletion mutants

*R. sphaeroides* deletions strains 2.4.1ΔRSP_1090, 2.4.1ΔRSP_1091-1087, 2.4.1ΔRSP_1096-1097, 2.4.1ΔRSP_3242 and 2.4.1ΔRSP_2710 were generated by transferring the respective suicide plasmid pPHU2.4.1RSP_1090::Km, pPHU2.4.1RSP_1091-1087::Km, pPHU2.4.1ΔRSP_1096-1097::Km, pPHU2.4.1ΔRSP_3242::Km and pPHU2.4.1ΔRSP_2710::Km (Table 1 and 2) into *R. sphaeroides* 2.4.1. Knockout candidates were screened for insertion of the kanamycin cassette into the chromosome by homologous recombination. For construction of pPHU2.4.1RSP_1090::Km parts of the gene RSP_1090 together with upstream and downstream regions were amplified by PCR using the oligonucleotides 2.4.1RSP1090_knockout-up_EcoRI, 2.4.1RSP1090_knockout-up_PstI, 2.4.1RSP1090_knockout-down_PstI and 2.4.1RSP1090_knockout-down_SphI (Table S2). Using the same strategy, parts of RSP_1091-1087 operon together with RSP_1091 upstream and RSP_1087 downstream regions were amplified by PCR using the oligonucleotides 2.4.1RSP_1091_knockout-up_EcoRI, 2.4.1RSP_1091_knockout-up_PstI, 2.4.1RSP_1087_knockout-down_PstI and 2.4.1RSP1087_knockout-down_SphI (Table S2). For construction of pPHU2.4.1RSP_1096-1097::Km RSP_1096-1097 together with upstream and downstream regions were amplified by PCR using the oligonucleotides 2.4.1RSP1096/97_knockout-up_EcoRI, 2.4.1RSP1096/97_knockout-up_PstI, 2.4.1RSP1096/97_knockout-down_PstI and 2.4.1 RSP1096/97_knockout-down_SphI (Table S2). For deletion of RSP_3242, part of RSP_3242 together with upstream and downstream regions were amplified by PCR using the oligonucleotides 2.4.1RSP3242_knockout-up_EcoRI, 2.4.1RSP3242_knockout-up_PstI, 2.4.1RSP3242_knockout-down_PstI and 2.4.1RSP3242_knockout-down_SphI (Table S2). RSP_2710 together with upstream and downstream regions were amplified by PCR using the oligonucleotides 2.4.1RSP2710_knockout-up_EcoRI, 2.4.1RSP2710_knockout-up_PstI, 2.4.1RSP2710_knockout-down_PstI and 2.4.1 RSP2710_knockout-down_SphI (Table S2). The obtained PCR fragments were cloned into pPHU281 [35] using the appropriate restriction endonucleases. Then the kanamycin cassette obtained from plasmid pUC4K [36] was inserted into the PstI restriction site to obtain the plasmids pPHU2.4.1RSP_1090::Km, pPHU2.4.1RSP_1091-1087::Km, pPHU2.4.1Δ1096-1097::Km, pPHU2.4.1ΔRSP_3242::Km and pPHU2.4.1ΔRSP_2710::Km. The plasmids were transferred into *E. coli* strain S17-1 [37] and mobilized into *R. sphaeroides* strains by biparental conjugation. Conjugants were selected on malate minimal medium agar plates containing 25 µg ml^-1^ kanamycin. PCR analyses of chromosomal DNA isolated from kanamycin resistant and tetracycline sensitive conjugants were carried out to confirm the double crossover event of the kanamycin cassette into the *R. sphaeroides* chromosome. 

**Table 1:** Strains.

**Strains**	**Description**	**Source/Reference**
***E. coli***		
S17-1	*recA pro; hsdR*; RP4- 2- Tc::Mu-Km::tn7*; tra* ^+^; Km^r^; Sp^r^	[37]
JM109	*recA1 supE44 endA1 hsdR17 gyrA96 relA1 thi (lac–proAB)*	New England Biolabs
M15(pREP4)	*E. coli* strain M15 containing pREP4 plasmid encoding *lac* repressor in trans, Km^r^	Qiagen
M15(pREP4)pQE30_2.4.1*rpoE*	*E. coli* M15 harboring pQE30_2.4.1*rpoE*, Km^r^, Ap^r^	This study
M15(pREP4)pQE30_2.4.1*chrR*	*E. coli* M15 harboring pQE30_2.4.1*chrR*, Km^r^, Ap^r^	This study
***R. sphaeroides***		
2.4.1	Wild type	[40]
2.4.1pPHU*phrAlacZ*	2.4.1 harboring pPHU*phrAlacZ*, Tc^r^	[15]
2.4.1pRK415	Wild type harboring pRK415, Tc^r^	This study
2.4.1Δ*RSP*_*1090*	2.4.1RSP*_1090*::Km^r^ cassette, Km^r^	This study
2.4.1Δ*RSP*_*1090*pRK415	2.4.1Δ*RSP*_*1090* harboring pRK415, Km^r^, Tc^r^	This study
2.4.1Δ*RSP*_*1091-1087*	2.4.1RSP*_1091-1087*::Km^r^ cassette, Km^r^	This study
2.4.1Δ*RSP*_*1096-1097*	2.4.1RSP*_1096-1097*::Km^r^ cassette, Km^r^	This study
2.4.1Δ*RSP*_*2710*	2.4.1RSP*_2710*::Km^r^ cassette, Km^r^	This study
2.4.1Δ*RSP*_*3242*	2.4.1RSP*_3242*::Km^r^ cassette, Km^r^	This study
2.4.1Δ*RSP*_*1090*pRK*RSP*_*1090*	2.4.1Δ*RSP*_*1090* harboring pRK*RSP*_*1090*, Km^r^, Tc^r^	This study
2.4.1Δ*RSP1090*pPHU*phrAlacZ*	2.4.1Δ*RSP*_*1090* harboring pPHU*phrAlacZ*, Km^r^,Tc^r^	This study
2.4.1Δ*RSP1090*pPHU*phrAlacZ*pBBR_*RSP*_*1090*	2.4.1Δ*RSP*_*1090* pPHU*phrAlacZ* harboring pBBR_*RSP*_*1090*, Km^r^,Tc^r^, Gm^r^	This study
2.4.1Δ*RSP*_*1091-1087*pRK*RSP*_*1090*	2.4.1Δ*RSP*_*1091-1087* harboring pRK*RSP*_*1090*, Km^r^, Tc^r^	This study
2.4.1Δ*RSP*_*1091-1087*pRK*RSP*_*1091*	2.4.1Δ*RSP*_*1091-1087* harboring pRK*RSP*_*1091*, Km^r^, Tc^r^	This study
2.4.1Δ*RSP*_*1091-1087*pRK*RSP*_*1091-1090*	2.4.1Δ*RSP*_*1091-1087* harboring pRK*RSP*_*1091-1090*, Km^r^, Tc^r^	This study
2.4.1Δ*RSP*_*1091-1087*pRK*RSP*_*1091-1087*	2.4.1Δ*RSP*_*1091-1087* harboring pRK*RSP*_*1091-1087*, Km^r^, Tc^r^	This study
TF18	*rpoEchrR* mutation in 2.4.1, Tp^r^	[41]
TF18pRK415	TF18 harboring pRK415, Tp^r^, Tc^r^	This study
TF18pPHU*phrAlacZ*	TF18 harboring pPHU*phrAlacZ*, Tp^r^, Tc^r^	[15]
TF18pPHU*phrAlacZ* pBBR_2.4.1*rpoEchrR*	TF18 pPHU*phrAlacZ* harboring pBBR_2.4.1*rpoEchrR* Tp^r^, Tc^r^, Gm^r^	This study
2.4.1Δ*RSP*_*1096-1097*pPHU*phrAlacZ*	2.4.1Δ*RSP*_*1096-1097* harboring pPHU*phrAlacZ*, Tp^r^, Tc^r^	This study
2.4.1Δ*RSP*_*2710*pPHU*phrAlacZ*	2.4.1Δ*RSP*_*2710*harboring pPHU*phrAlacZ*, Tp^r^, Tc^r^	This study
2.4.1Δ*RSP*_*3242*pPHU*phrAlacZ*	2.4.1Δ*RSP*_*3242* harboring pPHU*phrAlacZ*, Tp^r^, Tc^r^	This study
TF18pRK2.4.1*rpoEchrR*	TF18 harboring pRK2.4.1*rpoEchrR*, Tp^r^, Tc^r^	[42]

**Table 2:** Plasmids.

**Plasmids**	**Description**	**Source/Reference**
pPHU281	Tc^r^, suicide vector for *R. sphaeroides*, Tc^r^	[35]
pUC4K	Km^r^, source of Km^r^ cassette	[36]
pSUP202	Tc^r^, Km^r^, Suicide vector used for Tn5 mutagenesis	[37]
pRK415	Tc^r^	[43]
pBBR1MCS-5	Gm^r^	[44]
pPHU*phrAlacZ*	pPHU234 with *phrA* upstream-region, Tc^r^	[15]
pPHU2.4.1*RSP*_*1090*::Km^r^	pPHU281 with Km^r^ cassette, flanked by the up- and downstream region of RSP_1090	This study
pPHU2.4.1*RSP*_*RSP*_*1091-1087*::Km^r^	pPHU281 with Km^r^ cassette, flanked by the upstream region of RSP_1091	This study
	and downstream region of RSP_1087	
pPHU2.4.1*RSP*_*1096-1097*::Km^r^	pPHU281 with Km^r^ cassette, flanked by the up- and downstream region of RSP_1096-1097	This study
pPHU2.4.1*RSP*_*2710*::Km^r^	pPHU281 with Km^r^ cassette, flanked by the up- and downstream region of RSP_2710	This study
pPHU2.4.1*RSP*_*3242*::Km^r^	pPHU281 with Km^r^ cassette, flanked by the up- and downstream region of RSP_3242	This study
pRK*RSP*_*1090*	pRK415 harboring a 0.8 kb fragment containing RSP_1090 flanked by the 64 bp upstream region of RSP_1091 and 7 bp downstream region of RSP_1090	This study
pRK2.4.1*rpoEchrR*	pRK415 harboring a 1.6 kb fragment containing 2.4.1 *rpoEchrR* flanked by the 241 bp upstream and 158 bp downstream regions	[42]
pRK2.4.1RSP_1091	pRK415 harboring a 1.4 kb fragment containing RSP_1091 flanked by the 97 bp upstream region of RSP_1090 and 24 bp downstream region of RSP_1091	This study
pRK2.4.1RSP_1091-1090	pRK415 harboring a 2.1 kb fragment containing entire sequence of RSP_1091 and RSP_1090 flanked by the 97 bp upstream region of RSP_1091 and 19 bp downstream of RSP_1090	This study
pRK2.4.1RSP_1091-1087	pRK415 harboring a 4.6 kb fragment containing entire sequence of RSP_1091-1087 flanked by 99 bp upstream of RSP_1091 and 78 bp downstream of RSP_1087	This study
pBBR2.4.1*RSP*_*1090*	pBBR1MCS-5 harboring a 0.8 kb fragment containing RSP_1090 flanked by the 64 bp upstream region of RSP_1091 and 7 bp downstream region of RSP_1090	This study
pBBR_2.4.1*rpoEchrR*	pBBR1MCS-5 harboring a 1.6 kb fragment containing 2.4.1 *rpoEchrR* flanked by the 241 bp upstream and 158 bp downstream regions	This study
pQE30_2.4.1*rpoE*	pQE30 harboring the entire *rpoE* gene lacking the first codon	This study
pQE30_2.4.1*chrR*	pQE30 harboring the entire *chrR* gene lacking the first codon	This study
pQE30	Ap^r^, vector used for protein overexpression in *E. coli* M15(REP4)	Qiagen
*pDrive* cloning vector	Ap^r^; Km^r^	Qiagen

### Complementation of the *R. sphaeroides* RSP_1090 and RSP_1091-1087 deletion mutants

For complementation of strain 2.4.1ΔRSP_1090 and 2.4.1ΔRSP_1091-1087 with RSP_1090 a 821 bp PCR fragment, containing the entire gene along with 64 bp of the upstream region of RSP_1091, including the RpoE dependent RSP_1091 promoter, and 7 bp downstream of the last RSP_1090 codon, was amplified using the oligonucleotides 2.4.1RSP1090com_up_SigE1091 and 2.4.1RSP1090com_down (Table S2). The obtained PCR fragment was cloned into the *pDrive* vector (Qiagen). Digestion of the *pDrive* vector, containing the insert, with PstI and XbaI followed by cloning with the same restriction sites into plasmid pRK415 yielded plasmid pRK_RSP_1090. This plasmid was subsequently transformed into *E. coli* S17-1 and conjugated with strain 2.4.1ΔRSP_1090 to obtain the complemented strain 2.4.1ΔRSP_1090pRK_RSP_1090. Cloning of the PCR fragment with the same restriction enzymes into the vector pBBR1MCS-5 yielded plasmid pBBR2.4.1_RSP_1090.

The same strategy was applied for complementation of 2.4.1ΔRSP_1091-1087 with RSP1091, RSP_1090, RSP_1091-1090 and RSP_1091-1087. For complementation with RSP_1091, a 1.4 kb PCR fragment, containing the entire sequence of RSP_1091, along with 97 bp of the upstream region of RSP_1091 and 24 bp downstream of the last RSP_1091 codon, was amplified using the oligonucleotides 2.4.1RSP1091com_up_KpnI and 2.4.1RSP1091com_dn_XbaI (Table S2). For complementation with RSP_1091-1090, a 2.1 kb PCR fragment, containing the entire sequence of both the genes, RSP_1090 and RSP1091, along with 97 bp of the upstream region of RSP_1091 and 19 bp downstream of the last RSP_1090 codon, was amplified using the oligonucleotides 2.4.1RSP1091com_up_KpnI and 2.4.1RSP1090com_dn_XbaI (Table S2). Finally, for complementation with RSP_1091-1087 a 4.6 kb PCR fragment, containing the entire region of RSP_1091-1087 along with 99 bp of the upstream region of RSP_1091 and 78 bp downstream of the RSP_1087, was amplified using the oligonucleotides 2.4.1RSP1091-87com_up and 2.4.1RSP1091-87com_down (Table S2). The obtained PCR fragments were cloned into the *pDrive* vector (Qiagen). Digestion of the *pDrive* vector with KpnI and XbaI was followed by cloning with the same restriction sites into plasmid pRK415 yielding the strains 2.4.1ΔRSP_1091-1087pRK2.4.1RSP_1091, 2.4.1ΔRSP_1091-1087pRK2.4.1RSP_1091-1090, and 2.4.1ΔRSP_1091-1087pRK2.4.1RSP_1091-1087. 

### Construction of strains 2.4.1ΔRSP_1096-1097pPHU*phrAlacZ*, 2.4.1ΔRSP_2710pPHU*phrAlacZ*, 2.4.1ΔRSP_3242pPHU*phrAlacZ*, and TF18pPHU*phrAlacZ*

For construction of the strains 2.4.1ΔRSP_1096-1097pPHU*phrAlacZ*, 2.4.1ΔRSP_2710pPHU*phrAlacZ*, 2.4.1ΔRSP_3242pPHU*phrAlacZ* and TF18pPHU*phrAlacZ* the plasmid pPHU*phrAlacZ* was transferred to strains 2.4.1ΔRSP_1096-1097, 2.4.1ΔRSP_2710, 2.4.1ΔRSP_3242 and TF18 by biparental conjugation. 

### Construction of strains 2.4.1ΔRSP_1090pPHU*phrAlacZ*, 2.4.1ΔRSP_1090pPHU*phrAlacZ*pBBR2.4.1_RSP_1090 and TF18pPHU*phrAlacZ*pBBR_2.4.*1rpoEchrR*

For construction of strain 2.4.1ΔRSP_1090pPHU*phrAlacZ* the plasmid pPHU*phrAlacZ* was transferred to strain 2.4.1ΔRSP_1090 by biparental conjugation. Transfer of plasmid pBBR2.4.1_RSP_1090 to strain 2.4.1ΔRSP_1090pPHU*phrAlacZ* yielded strain 2.4.1ΔRSP_1090pPHU*phrAlacZ*pBBR2.4.1_RSP_1090. For construction of plasmid pBBR2.4.1*rpoEchrR* the plasmid pRK2.4.1*rpoEchrR* [38] was digested with EcoRI. The obtained 2.4.1*rpoEchrR* fragment was cloned into pBBR1MCS-5 and transformed in *E*. *coli* S17-1 and conjugated with strain TF18pPHU*phrAlacZ* yielding TF18pPHU*phrAlacZ*pBBR2.4.1*rpoEchrR*. 

### Construction and purification of recombinant proteins and production of antibodies

The *rpoE* (RSP_1092) and the *chrR* (RSP_1093) gene of *R. sphaeroides* 2.4.1 were PCR amplified, from the second to the last codon, using the oligonucleotides *rpo*E-A-4, *rpo*E-B542, *chr*R-A-4 and *chr*R-B-638. The PCR products were cloned into the pQE30 vector (Qiagen). Overexpression in *E. coli* M15 (pREP4) cells was performed as described earlier [39]. Purification using nickel-nitriloacetic-acid (Ni-NTA) agarose was performed under denaturing conditions in accordance with the manufacturer’s instructions (Qiagen). For production of polyclonal antibodies raised against His_6_-RpoE, 750 µg of recombinant protein were separated by SDS-PAGE and stained with ice cold 3 M potassium chloride solution. Protein bands were cut out of the gel, production of antibodies in rabbits was performed by BioGenes, Berlin. Antibodies were purified by using CNBr-activated sepharose (GE Healthcare, Munich) coupled with His_6_-RpoE. For production of polyclonal antibodies in rabbits raised against His_6_-ChrR, purified recombinant protein was sent to Davids Biotechnologie in Regensburg. Antibodies were purified by using affinity purification via western blotting. The His_6_-ChrR protein bands were excised from the membrane and incubated with serum for three hours. After washing the membrane with 1xTBS, the bound antibody was eluted with acidic glycine buffer and immediately neutralized with 1M Tris (pH 8.0). 

### Western blot experiments

For Western blot experiments *R. sphaeroides* cultures were grown under non-stress or ^1^O_2_ stress conditions as described above. To determine stability of RpoE and ChrR chloramphenicol was added in a final concentration of 500 µg ml^-1^. Aliquots were taken at different time points, rapidly cooled and ice-cold trichloroacetic acid (10% final concentration) was added and incubated on ice for one hour. For precipitation of the protein samples were centrifuged at 13.000 rpm for 10 minutes. The supernatant was aspirated and the pellet washed twice with ice-cold 100% acetone. After evaporation of residual acetone, the cell pellet was suspended in 1 fold tris buffer saline (1xTBS) with 0.05% tween-20. Equal amounts of total protein (300 µg) were separated on a 12% PAA-SDS gel and transferred to a nitrocellulose membrane (Whatman). Proteins were stained and fixed with Ponceau S (Sigma Aldrich), destained with sodium hydroxide and the membrane was blocked at room temperature for 1 hour in blocking buffer (1xTBS) containing 5% (w/v) of milk powder (Roth). After blocking, the purified primary antibodies, α-RpoE or α-ChrR, diluted 1:5.000 in blocking buffer, were added to the membrane and incubated for 3 hours. After washing the membrane 3 times for 5 min in 1xTBS buffer, the secondary antibody (anti-rabbit IgG conjugated with peroxidases, produced in goat, Sigma Aldrich) was added (diluted 1:15.000 in blocking buffer) and the membrane further incubated for 2 hours at room temperature. The membrane was washed 3 times with 1xTBS for 5 minutes. The washing step was repeated 2 times. Western blots were developed using the lumi-light western blotting substrate 1 and 2 (Roche).

## Supporting Information

Figure S1 **The RSP_1091-1090 locus is well conserved in α-proteobacteria.** Gene neighborhood of the *R. sphaeroides* 2.4.1 gene RSP_1090 in selected genomes of the α-proteobacteria. Homologs of RSP_1090 were searched by using the BLAST option on the integrated microbial genome (IMG) website. The genes encoding the retrieved homologs of RSP_1090 were subsequently analyzed with respect to the homology of proteins encoded by adjacent genes. In bacteria a number of 337 genomes contained RSP_1090 with an upstream located homolog of RSP_1091. Amino acid identities to *R. sphaeroides* 2.4.1 proteins are indicated.
(PDF)

Table S1 **Inhibition zone diameters.** Sensitivity against ^1^O_2_ and organic peroxide (tBOOH) was tested for the *R. sphaeroides* wild type, strain 2.4.1Δ*RSP*_*1090* and strain TF18. For strains harbouring plasmid pRK415 and the constructs pRK*RSP*_*1090* and pRK_2.4.1*rpoEchrR*, also values for ^1^O_2_ and tBOOH inhibition zones were determined. The generation of ^1^O_2_ was achieved by applying 5µl of 10 mM methylene blue solution on filter discs placed on agar plates in the light. In the same manner 700 mM tBOOH was used, agar plates were incubated in the dark. In all cases the mean and standard deviation for three replicates are depicted. Mean values of three experiments are given, SD: standard deviation.
(DOC)

Table S2 **Oligonucleotides used throughout this study.**
(DOC)
