# Supplementary material for: DegS and RseP Homologous Proteases Are Involved in Singlet Oxygen Dependent Activation of RpoE in Rhodobacter sphaeroides
Source: PLoS One. 2013 Nov 5;8(11):e79520. doi: 10.1371/journal.pone.0079520 (PMC3818230; doi:10.1371/journal.pone.0079520)
Supplement: Figure S1 — The RSP_1091-1090 locus is well conserved in α-proteobacteria. Gene neighborhood of the R. sphaeroides 2.4.1 gene RSP_1090 in selected genomes of the α-proteobacteria. Homologs of RSP_1090 were searched by using the BLAST option on the integrated microbial genome (IMG) website. The genes encoding the retrieved homologs of RSP_1090 were subsequently analyzed with respect to the homology of proteins encoded by adjacent genes. In bacteria a number of 337 genomes contained RSP_1090 with an upstream located homolog of RSP_1091. Amino acid identities to R. sphaeroides 2.4.1 proteins are indicated. (PDF) [file pone.0079520.s001.pdf]

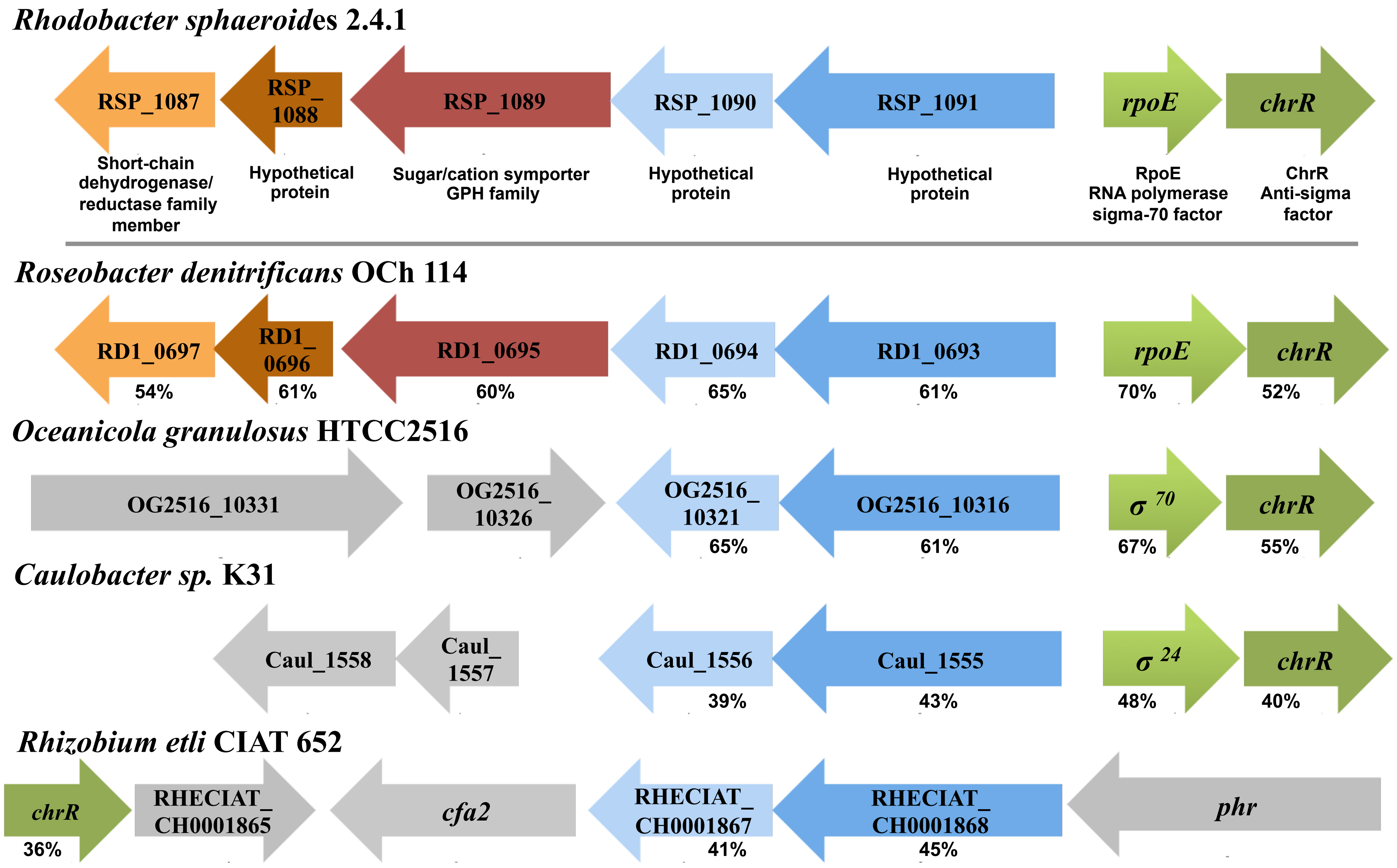
Rhodobacter sphaeroides 2.4.1
RSP_1087
RSP_1088
RSP_1089
RSP_1090
RSP_1091
rpoE
chrR
Short-chain dehydrogenase/ reductase family member
Hypothetical protein
Sugar/cation symporter GPH family
Hypothetical protein
Hypothetical protein
RpoE RNA polymerase sigma-70 factor
ChrR Anti-sigma factor
Roseobacter denitrificans OCh 114
RD1_0697
54%
RD1_0696
61%
RD1_0695
60%
RD1_0694
65%
RD1_0693
61%
rpoE
70%
chrR
52%
Oceanicola granulosus HTCC2516
OG2516_10331
OG2516_10326
OG2516_10321
65%
OG2516_10316
61%
σ 70
67%
chrR
55%
Caulobacter sp. K31
Caul_1558
Caul_1557
Caul_1556
39%
Caul_1555
43%
σ 24
48%
chrR
40%
Rhizobium etli CIAT 652
chrR
36%
RHECIAT_CH0001865
cfa2
RHECIAT_CH0001867
41%
RHECIAT_CH0001868
45%
phr

Figure S1
