# Supplementary material for: DegS and RseP Homologous Proteases Are Involved in Singlet Oxygen Dependent Activation of RpoE in Rhodobacter sphaeroides
Source: PLoS One. 2013 Nov 5;8(11):e79520. doi: 10.1371/journal.pone.0079520 (PMC3818230; doi:10.1371/journal.pone.0079520)
Supplement: Table S1 — Inhibition zone diameters. Sensitivity against 1O2 and organic peroxide (tBOOH) was tested for the R. sphaeroides wild type, strain 2.4.1ΔRSP_1090 and strain TF18. For strains harbouring plasmid pRK415 and the constructs pRKRSP_1090 and pRK_2.4.1rpoEchrR, also values for 1O2 and tBOOH inhibition zones were determined. The generation of 1O2 was achieved by applying 5µl of 10 mM methylene blue solution on filter discs placed on agar plates in the light. In the same manner 700 mM tBOOH was used, agar plates were incubated in the dark. In all cases the mean and standard deviation for three replicates are depicted. Mean values of three experiments are given, SD: standard deviation. (DOC) [file pone.0079520.s002.doc]

| Strains | Inhibition zone (cm±SD) | |
| --- | --- | --- |
|  | ^1^O_2_ | tBOOH |
| 2.4.1 | 2.5±0.1 | 1.6±0.1 |
| 2.4.1pRK415 | 2.5±0.1 | 1.6±0.1 |
| 2.4.1Δ*RSP_1090* | 3.0±0.1 | 1.6±0.1 |
| 2.4.1Δ*RSP_1090*pRK415 | 3.1±0.1 | 1.8±0.1 |
| 2.4.1Δ*RSP_1090*pRK*RSP_1090* | 2.4±0.1 | 1.7±0.1 |
| TF18 | 3.0±0.1 | 2.6±0.1 |
| TF18pRK415 | 3.0±0.1 | 2.6±0.1 |
| TF18pRK_2.4.1*rpoEchrR* | 2.3±0.1 | 1.8±0.1 |
