# Supplementary material for: DegS and RseP Homologous Proteases Are Involved in Singlet Oxygen Dependent Activation of RpoE in Rhodobacter sphaeroides
Source: PLoS One. 2013 Nov 5;8(11):e79520. doi: 10.1371/journal.pone.0079520 (PMC3818230; doi:10.1371/journal.pone.0079520)
Supplement: Table S2 — Oligonucleotides used throughout this study. (DOC) [file pone.0079520.s003.doc]

| **Oligonucleotide** | **Sequence** | **Reference** |
| --- | --- | --- |
| RSP1097.KO.up.EcoRI | 5´- CCGGAATTCGGAACCTCGCCGATCCGAATC -3´ | This study |
| RSP1097.KO.up.PstI | 5´- CGCCTGCAGCCGCGATACCAGACCATGTGGG-3´ | This study |
| RSP1097.KO.dn.PstI | 5´- CGCCTGCAGGGTGACGCGCAATGAGAAGATC-3´ | This study |
| RSP1097.KO.dn.SphI | 5´- ACATGCATGCAGGTGATGGTGAGCCGCC-3´ | This study |
| Test.Out.RSP1097.up | 5´-GAACCTGCGTTCCACCGG-3´ | This study |
| Test.Out.RSP1097.dn | 5´-CGCGCTGACGGCGATCTG-3´ | This study |
| Test.Inside.RSP1097.up | 5´- CACCCACATGGTCTGGTATC-3´ | This study |
| Test.Inside.RSP1097.dn | 5´- CATCTGCATCCCCACGAG -3´ | This study |
| RSP3242.KO.up.EcoRI | 5´- CCGAATTCGGATCGGCGCCTTGATCTGGCG -3´ | This study |
| RSP3242.KO.up.PstI | 5´- CGCCTGCAGCCGTCACCTCGATGAAGACGAC-3´ | This study |
| RSP3242.KO.dn.PstI | 5´CGCCTGCAAGGCAAGGGCGTGATGCTGATGAAG-3 | This study |
| RSP3242.KO.dn.SphI | 5´- ACATGCATGCAACCCGTGGAGGTAGACCC -3´ | This study |
| Test.Out.RSP3242.up | 5´- CGCGCTGACAGTTCCGAG -3´ | This study |
| Test.Out.RSP3242.dn | 5´- CATGCCCGAAGCGCCGTC -3´ | This study |
| Test.Inside.RSP3242.up | 5´- GTCGAAGGAGTCCACGCC -3´ | This study |
| Test.Inside.RSP3242.dn | 5´- CATCGCCGAGGCCAGATC -3´ | This study |
| TestInsideRSP2710.up | 5´- GGCATCCATGCCGAGGTC -3´ | This study |
| Test.Inside.RSP2710.dn | 5´- GAGGATCGGAACCGGGAAG-3´ | This study |
| RSP2710KO.up.EcoRI | 5´- CCGGAATTCGCCGCAATCCATCGTTCATGCC -3´ | This study |
| RSP2710.KO.up.PstI | 5´- CGCCTGCAGCGGAAAGAGCGACGACGAATGC-3´ | This study |
| RSP2710.KO.dn.PstI | 5´- CGCCTGCAGGGGCGATTTGGCGTTGTGAAGG -3´ | This study |
| RSP2710.KO.dn.SphI | 5´- ACATGCATGCGAACAGGCCCGAATCCGC-3´ | This study |
| Test.Out.RSP2710.up | 5´- CAGCTCCTGATGCGGACG -3´ | This study |
| Test.Out.RSP2710.dn | 5´- GCTTGTCGTCCTTGATGCG -3´ | This study |
| RSP0197.KO.up.EcoRI | 5´- CCGGAATTCGGGGATGCCGTGACGATGGTGG -3´ | This study |
| RSP0197.KO.up.PstI | 5´- CGCCTGCAGCGACGTTTGCAAGGATTATCGTC-3 | This study |
| RSP0197.KO.dn.PstI | 5´- CGCCTGCAGGGGCGATTTGGCGTTGTGAAGG -3´ | This study |
| RSP0197.KO.dn.SphI | 5´- ACATGCATGCTTGTCGACCTCGTCGATATAG -3´ | This study |
| RSP1098_up | 5´- GTGAAGCCGCTGGAGATTC -3´ | This study |
| RSP1098_down | 5´- CCGCCAGGCATAATCCTC -3´ | This study |
| Km-forward | 5´- CATGAACAATAAAACTGTCTGC-3´ | This study |
| Km-reverse | 5´- GGATCAGATCACGCATCTTC-3´ | This study |
| Sp-forward | 5´- CTGCAGGGTGATTGATTGAGCAAG-3´ | This study |
| Sp-reverse | 5´- CTGCAGGGTGATTGATTGAGCAAG-3´ | This study |
| 2.4.1RSP1091-87com_up | 5´- GGCCAGTCTGGATCAGGC-3´ | This study |
| 2.4.1RSP1091-87com_down | 5´- GGTCCCGTCGGGGCAACG-3´ | This study |
| 2.4.1RSP1091com_up_KpnI | 5´- GGCGGTACCGGCCAGTCTGGATCAGGCGGG-3´ | This study |
| 2.4.1RSP1091com_dn_XbaI | 5´- GGCTCTAGACATGATCGACCCACATGACGCGC-3´ | This study |
| 2.4.1RSP1090com_dn_XbaI | 5´- GGCTCTAGAGCACTCACCGCGTGACGTCTTG-3´ | This study |
